# Factors influencing the long-term-success of the XEN gel stent

**DOI:** 10.1007/s00417-025-07104-0

**Published:** 2026-01-13

**Authors:** Caroline J. Wenzel, Daniel A. Wenzel, Christina Pagonidou, Vasyl Druchkiv, Emil Nasyrov, Bogomil Voykov

**Affiliations:** 1 Centre for Ophthalmology, University Hospital Tübingen, Elfriede-Aulhorn-Str. 7, 72076 Tübingen, Germany; 2 Department of Research and Development, Clinica Baviera-AIER Eye Group, Valencia, Spain; 3 Department of Ophthalmology, University Clinic Hamburg-Eppendorf, University Medical Center Hamburg-Eppendorf, Hamburg, Germany; 4 Augencentrum Südwest, Stuttgart, Germany

**Keywords:** Glaucoma, XEN gel stent, Risk factor, Minimally-invasive bleb surgery

## Abstract

**Purpose:**

The XEN gel stent is a minimally invasive surgical device for patients with open-angle glaucoma, developed to reduce complications associated with traditional filtration surgery. This study aimed to identify clinical factors that influence long-term surgical success, particularly in the context of postoperative conjunctival scarring.

**Methods:**

In this retrospective study, 773 eyes of patients with glaucoma treated at a tertiary care center underwent implantation of the XEN gel stent. The primary outcome was long-term surgical success, defined as a reduction in intraocular pressure of at least 20% and achieving a target pressure of 18, 15, or 12 mmHg, either without (complete) or with (qualified) medication. Secondary outcomes included intraocular pressure reduction, change in best-corrected visual acuity and medication use. Potential risk factors were analysed using Kaplan-Meier survival curves and multivariable Cox regression.

**Results:**

At 24 months, complete and qualified success for a target pressure of 18 mmHg was achieved in 50% and 64% of cases, respectively; at 72 months, in 35% and 54%. Visual acuity remained stable. Female sex, mitomycin C dose, moderately elevated baseline pressure, and lower pressure on the first postoperative day were associated with significantly higher success rates (*p* < 0.05). Glaucoma subtype and previous incisional glaucoma surgery had no significant effect.

**Conclusion:**

Sex, lower antimetabolite dose and intraocular pressure values before and shortly after surgery are significant predictors of long-term success following XEN gel stent implantation.

## Key messages

**What is known:**

• XEN gel stent is a minimally-invasive bleb forming device reducing intraocular pressure effectively with a favorable risk profile. However, bleb fibrosis remains the main factor limiting long-term success.

**What is new:**

• Sex, antimetabolite dose and perioperative intraocular pressure were identified as predictors for long-term surgical success of the XEN gel stent.

• Knowledge of these risk factors is crucial for developing future therapeutic approaches to improve surgical outcomes and for optimal patient selection.

## Introduction

Intraocular pressure (IOP) is the only modifiable risk factor of glaucoma progression [1]. When the target IOP cannot be achieved with topical medications, or if the patients experience adverse effects, surgical procedures are required to prevent further glaucomatous damage of the retinal ganglion cells and optic nerve. Minimally invasive bleb surgery (MIBS) with the XEN gel stent (AbbVie Inc., North Chicago, Illinois, USA) has been developed to reduce IOP with a better safety profile compared to trabeculectomy [2].

The XEN gel stent is a 6.00 mm-long gelatin tube designed to be implanted *ab interno* without conjunctival dissection. It drains aqueous humor into the subconjunctival space, creating a filtering bleb. To minimize the risk of postoperative scarring, intraoperative application of the cytostatic agent mitomycin C (MMC) is routinely used. Nevertheless, a significant proportion of patients with a XEN gel stent require surgical bleb revisions in the postoperative period due to scarring that compromises the drainage function.

The aim of this study is to analyse the long-term success of the XEN gel stent and identify factors that influence the likelihood of success.

## Materials and methods

This retrospective study included consecutive eyes receiving XEN45 gel stent implantation between December 2015 and April 2023 at a tertiary care center. All procedures were performed by a single experienced glaucoma surgeon.

All patients with primary or secondary open-angle glaucoma who received XEN 45 Gel Stent implantation during the study period were eligible for inclusion. Patients who received a larger version of the XEN Gel Stent with a 63 μm lumen were excluded.

The study followed the Declaration of Helsinki and was approved by the ethics committee of the University of Tübingen (project number: 423/2021B02).

### Outcome measures

Primary outcomes were rates of complete and qualified success. Secondary outcomes included changes in IOP, BCVA and medication use. In accordance with the World Glaucoma Association guidelines [3] recommendations, surgical success was evaluated using both IOP reduction and absolute postoperative IOP thresholds. Success was defined as an IOP reduction ≥ 20% and achieving target values of ≤ 18, ≤15, or ≤ 12 mmHg, without medication (complete success) or with and without medication (qualified success). Hypotony was defined as IOP < 6 mmHg. Failure included any additional glaucoma surgery or loss of light perception. Bleb revisions and needlings were not considered as failures and did not lead to censoring in the Kaplan–Meier survival analysis.

Each active agent in combination therapy was counted individually.

### Surgical technique

#### XEN gel stent implantation

Surgery was performed as previously described [4]. Under topical anesthesia with oxybuprocaine eye drops, two points at 3 mm distance from the limbus were marked in the superonasal quadrant. Initially, we followed the common expert recommendations for the dose of MMC, which was 20 µg (0.1 ml of 0.2 mg/ml MMC). Since we repeatedly observed avascular filtering blebs in the early postoperative period, indicating that the initial MMC concentration may have been higher than necessary, we made a first MMC dose reduction to 10 µg (0.05 ml of 0.2 mg/ml MMC) at 14 months after the first stent implantation at our centre, followed by an additional dose reduction to 5 µg (0.025 ml of 0.2 mg/ml MMC) 3 months later. The 5 µg dose was then continued. Therefore, MMC doses depended on the time at which the surgery was performed, and not on the surgeon’s decision.

A 20G side-port knife was used for the main incision 1 mm inferotemporally into the clear cornea and a smaller incision at the 11 or 2 o’clock position for the right or left eye. The anterior chamber was filled with Healon PRO Ophthalmic Viscoelastic Device (OVD; Johnson & Johnson, NJ, USA). The XEN45 injector was inserted through the main incision under gonioscopic control. The needle was advanced through the sclera and into the subconjunctival space between the two marked points. The stent was released, and the injector was withdrawn. After verification of the correct position of the stent, the viscoelastic was flushed out with balanced salt solution. Cefuroxime was injected into the anterior chamber.

### Pre- and postoperative management

Postoperatively, moxifloxacin was given for one week; unpreserved dexamethasone was tapered over five weeks. Follow-ups were recorded on days 1 and 2, then at months 3, 6, 9, and 12, and semiannually thereafter up to 72 months. In cases of inadequate pressure control or bleb scarring, needling or incisional revision was preferred over resuming topical therapy.

### Statistical analysis

Demographic and clinical data were summarized using descriptive statistics. IOP values were log-transformed, and a mixed-effects regression model was applied with subject as a random effect and time as a fixed effect. Since BCVA values showed a right-skewed distribution, with many observations clustered around 0 and few extreme values, a robust mixed-effects regression model was applied to obtain stable estimates in the presence of outliers and non-normal residuals. Changes in the number of medications were evaluated using a Poisson mixed-effects regression model. Kaplan–Meier survival curves were generated for categorical variables. For continuous predictors, Kaplan–Meier curves were plotted after categorising values into quantile-based groups using the 25th and 75th percentiles (Q1, Q3): low (< Q1), middle (Q1–Q3), and high (> Q3). These visualisations are exploratory and intended to illustrate unadjusted survival patterns, complementing the multivariable Cox regression results. Potential risk factors associated with surgical success were assessed using multivariable Cox proportional hazards regression models.

Data were reported as frequency (percentage), mean (standard deviation [SD]), median (95% confidence interval [95% CI]) or median (range), as appropriate. A p value < 0.05 was considered statistically significant.

## Results

### Characteristics of the study population

The study included 773 eyes of 596 patients which received XEN45 gel stent implantation between December 2015 and April 2023. In 90 (11.6%) of the eyes, XEN implantation was combined with cataract surgery. More than half of the eyes (405 eyes, 52.4%) had no previous glaucoma surgery or laser treatment.

Baseline characteristics and glaucoma subtypes are presented in Table 1.

**Table 1:** Baseline characteristics of the study population and intraoperative MMC dosing

Demographic and clinical characteristics of the study population		
Characteristic	*N*	%
Number of eyes (right/left)	773 (364/409)	100 (47.1/52.9)
Age in years, mean ± SD	66.0 ± 16.0	
Gender (female/male)	352/421	45.5/54.5
Lens status (phakic/pseudophakic)	388/385	50.2/49.8
Intraoperative MMC concentration	***N*** **(female/male)**	**%**
MMC 20 µg	114 (46/68)	14.7
MMC 10 µg	51 (25/26)	6.6
MMC 5 µg	582 (268/314)	75.3
No MMC (5-FU 5 mg)	26 (12/14)	3.4
Prior glaucoma surgery or laser treatment		
Procedure	***N***	**%**
Selective laser trabeculoplasty	78	10.1
Diode laser cyclophotocoagulation	105	13.6
Trabeculectomy	69	8.9
Canaloplasty	21	2.7
Deep sclerectomy	3	0.4
Trabeculotomy	46	5.9
Ahmed glaucoma valve	14	1.8
Cyclocryocoagulation	32	4.1
Glaucoma subtypes		
Subtype	***N***	**%**
Primary open-angle glaucoma	431	55.8
Pseudoexfoliative glaucoma	160	20.7
Uveitic glaucoma	65	8.3
Pigmentary glaucoma	44	5.7
Congenital glaucoma	20	2.6
Normal tension glaucoma	20	2.6
Neovascular glaucoma	15	1.9
Traumatic glaucoma	8	1.0
Aphakic glaucoma	4	0.5
Glaucoma secondary to iridocorneal endothelial syndrome	2	0.3
Glaucoma secondary to iris melanoma	2	0.3
Uveitis-glaucoma-hyphema syndrome	2	0.3

### Overall success rates

Complete success with a target IOP ≤ 18 mmHg, was observed in 58%, 50%, 40% and 35% at 12, 24, 60 and 72 months. Qualified success was achieved in 68%, 64%, 55% and 54% of eyes at 12, 24, 60 and 72 months.

For a stricter target IOP of ≤ 15 mmHg, complete success was achieved in 45%, 39%, 28% and 26% at 12, 24, 60 and 72 months. Qualified success for the same target was achieved in 52%, 47%, 34% and 31% at 12, 24, 60 and 72 months.

**Fig. 1:**
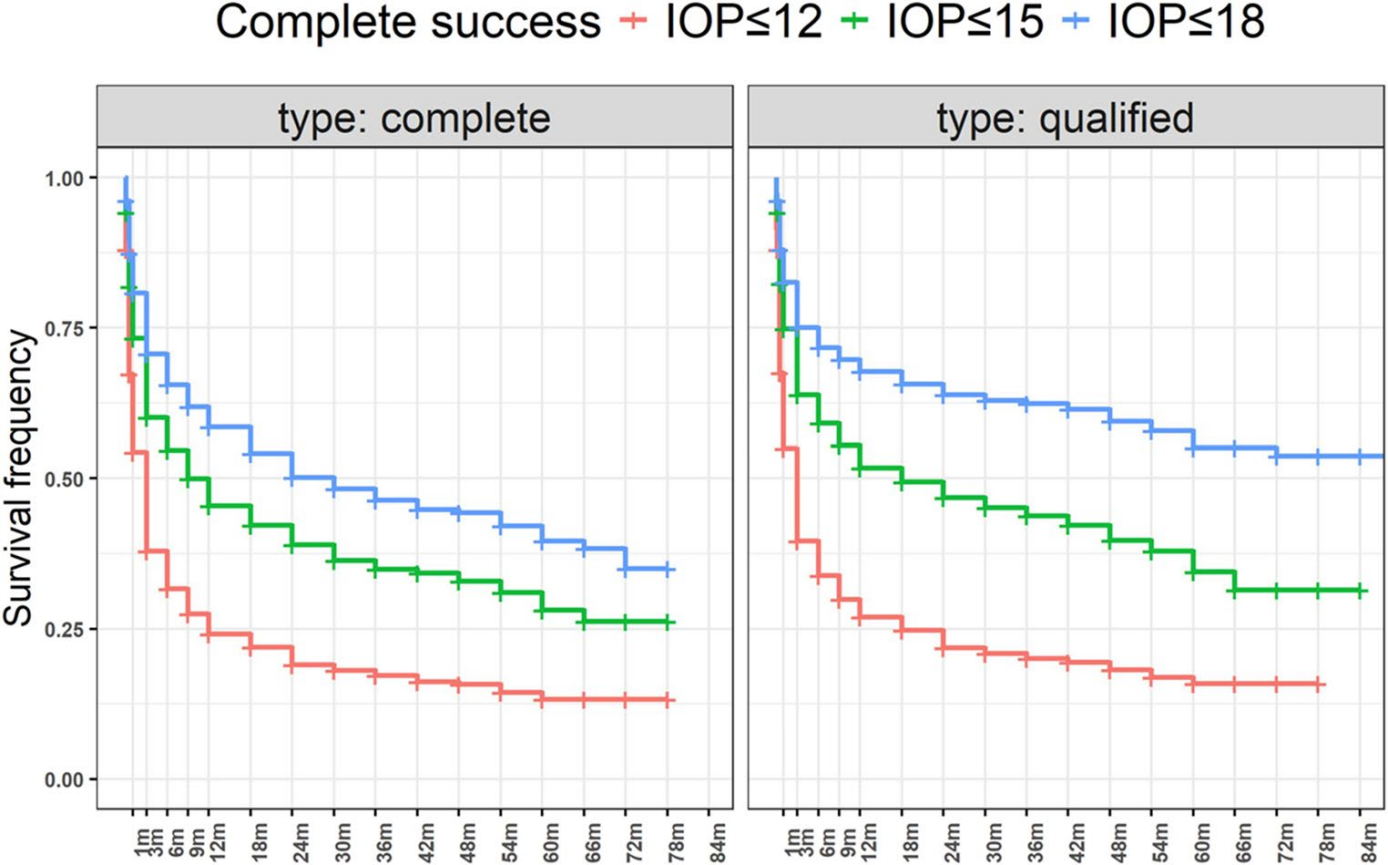
Overall success rates depicted in Kaplan-Meier curves. Complete success is shown in the left graph, qualified success in the right graph

For target IOP ≤ 12 mmHg, complete success was observed in 24%, 19%, 13% and 13% at 12, 24, 60 and 72 months. Qualified success was achieved in 27%, 22%, 16% and 16% at 12, 24, 60 and 72 months. The Kaplan–Meier curves are illustrated in Fig. 1.

### Development of IOP control, BCVA and medication

The median (± SD) IOP was reduced significantly from 25.0 (21.0–30.5.0.5) mmHg at baseline to 14.0 (12.0–17.0) mmHg after 12 months and remained at 14.0 (12.0–17.0) mmHg after 24 months and 14.0 (11.0–17.0) mmHg after 72 months (*p* < 0.001, respectively).

The median IOP reduction from baseline was 42.9% (26.6–56.7%), 40.6% (25.0–55.8%), 40.8% (27.0–57.7%), 44.4% (23.7–61.4%), 37.9% (24.5–53.8%) and 38.9% (21.4–54.7%) after 12, 24, 36, 48, 60 and 72 months, respectively.

Mean BCVA (± SD) declined significantly from 0.24 (± 0.19) logMAR at baseline to 0.27 (± 0.19; *p* = 0.007) logMAR at 12 months and remained at 0.27 (± 0.19) logMAR at 24 months (*p* = 0.81). Mean BCVA was 0.3 (± 0.19) logMAR, 0.32 (± 0.26) logMAR, 0.33 (± 0.39) logMAR, and 0.32 (± 0.07) logMAR after 36, 48, 60 and 72 months, respectively (*p* > 0.05, respectively).

The median (95% CI) number of IOP-lowering drugs was significantly reduced at all postoperative time points decreasing from 3.0 (2.9; 3,2) at baseline to 0.4 (0.4; 0.5) at 12 months, 0.6 (0.5; 0.7) at 24 months, and 0.6 (0.5; 0.8), 0.9 (0.7; 1.0), 1.2 (1.0; 1.4), 1.2 (0.9; 1.5) at 36, 48, 60 and 72 months, respectively (*p* < 0.001 for each timepoint compared to baseline).

### Impact of risk factors on success rates

Multivariable Cox proportional hazards regression analysis revealed variations in success rates depending on specific risk factors. The hazard ratios and p values for each risk factor examined are reported in Table 2.

**Table 2:** Results of multivariable Cox regression model. The table presents hazard ratios (HRs) with 95% confidence intervals from the multivariable Cox regression model. For categorical variables with more than two levels, an additional row provides the overall p-value obtained from the type II ANOVA

Risk factors	HR (95% confidence interval)					
	Complete success with IOP reduction ≥ 20%			Qualified success with IOP reduction ≥ 20%		
Target IOP level	≤ 12 mmHg	≤ 15 mmHg	≤ 18 mmHg	≤ 12 mmHg	≤ 15 mmHg	≤ 18 mmHg
Male sex	1.32 [1.11, 1.58]**	1.24 [1.01, 1.52]*	1.14 [0.91, 1.42]	1.37 [1.14, 1.64]***	1.39 [1.12, 1.72]**	1.31 [1.01, 1.70]*
Age	0.99 [0.99, 1.00].	1.00 [0.99, 1.01]	1.00 [0.99, 1.01]	0.99 [0.99, 1.00]	1.00 [0.99, 1.01]	1.00 [0.99, 1.01]
Baseline IOP	1.01 [1.00, 1.02]	1.00 [0.98, 1.01]	0.98 [0.96, 0.99]**	1.01 [1.00, 1.02]	0.99 [0.98, 1.01]	0.96 [0.94, 0.98]***
Postoperative day one IOP	1.08 [1.07, 1.09]***	1.08 [1.06, 1.10]***	1.05 [1.04, 1.06]***	1.08 [1.07, 1.09]***	1.08 [1.07, 1.10]***	1.06 [1.04, 1.08]***
Antimetabolite dose^†^, ANOVA p value	**< 0.001**	**0**.**018**	0.155	**< 0.001**	**0**.**002**	0.138
MMC 10 µg	1.20 [0.82, 1.75]	1.40 [0.92, 2.11]	0.99 [0.61, 1.60]	1.12 [0.77, 1.64]	1.34 [0.87, 2.06]	0.93 [0.53, 1.64]
MMC 5 µg	0.74 [0.58, 0.95]*	0.87 [0.66, 1.14]	1.00 [0.74, 1.36]	0.67 [0.53, 0.86]**	0.75 [0.57, 1.00].	0.89 [0.63, 1.25]
5-FU	1.66 [1.02, 2.70]*	1.52 [0.89, 2.62]	1.93 [1.10, 3.39]*	1.65 [1.02, 2.69]*	1.49 [0.84, 2.64]	1.94 [1.02, 3.71]*
Pseudophakia	0.91 [0.75, 1.10]	0.91 [0.73, 1.14]	1.02 [0.81, 1.30]	0.91 [0.75, 1.10]	0.90 [0.72, 1.14]	1.00 [0.76, 1.32]
Previous glaucoma surgery	0.95 [0.76, 1.18]	1.03 [0.80, 1.32]	1.26 [0.96, 1.64].	0.87 [0.70, 1.09]	0.81 [0.62, 1.07]	1.07 [0.78, 1.48]
Glaucoma type‡, ANOVA p value	0.335	0.105	0.107	0.12	0.061	0.241
Pseudoexfoliative	0.83 [0.67, 1.04]	0.93 [0.72, 1.21]	1.00 [0.75, 1.34]	0.76 [0.61, 0.96]*	0.86 [0.65, 1.13]	0.95 [0.68, 1.34]
Pigmentary	1.06 [0.74, 1.52]	1.53 [1.04, 2.26]*	1.68 [1.12, 2.52]*	1.06 [0.74, 1.52]	1.53 [1.02, 2.30]*	1.63 [1.03, 2.59]*
Uveitic	0.93 [0.65, 1.33]	1.63 [1.12, 2.36]*	1.52 [1.01, 2.30]*	0.84 [0.58, 1.21]	1.47 [0.98, 2.20].	1.07 [0.63, 1.81]
Congenital	1.02 [0.54, 1.93]	1.78 [0.89, 3.56]	1.28 [0.60, 2.76]	1.13 [0.59, 2.15]	2.67 [1.32, 5.43]**	1.97 [0.85, 4.56]
Normal tension	0.73 [0.42, 1.26]	0.84 [0.44, 1.60]	1.07 [0.56, 2.04]	0.77 [0.45, 1.33]	0.97 [0.51, 1.85]	1.30 [0.66, 2.58]
Neovascular	0.58 [0.30, 1.11].	1.31 [0.67, 2.56]	2.00 [1.00, 4.01]*	0.57 [0.30, 1.08].	1.35 [0.69, 2.65]	1.94 [0.88, 4.27].

Statistically significant p values are marked in bold or coded as follows: ***p < 0.001; **p < 0.01; *p < 0.05; · *p* < 0.1;† reference category 20 µg ‡ reference category primary open-angle glaucoma

### Sex

Female patients demonstrated significantly higher success rates for the stricter IOP targets. Specifically, for a target IOP of ≤ 12 mmHg, complete success was achieved significantly more often in female eyes compared to male eyes (*p* = 0.006), as was qualified success (*p* = 0.002). For the IOP target of ≤ 15 mmHg, the rates of complete success did not differ significantly (*p* = 0.054), but the rate of qualified success was significantly higher in female eyes (*p* = 0.004).

However, no statistically significant differences between sexes were observed for the target IOP of ≤ 18 mmHg (*p* = 0.33 and *p* = 0.058 for complete and qualified success, respectively).

**Fig. 2:**
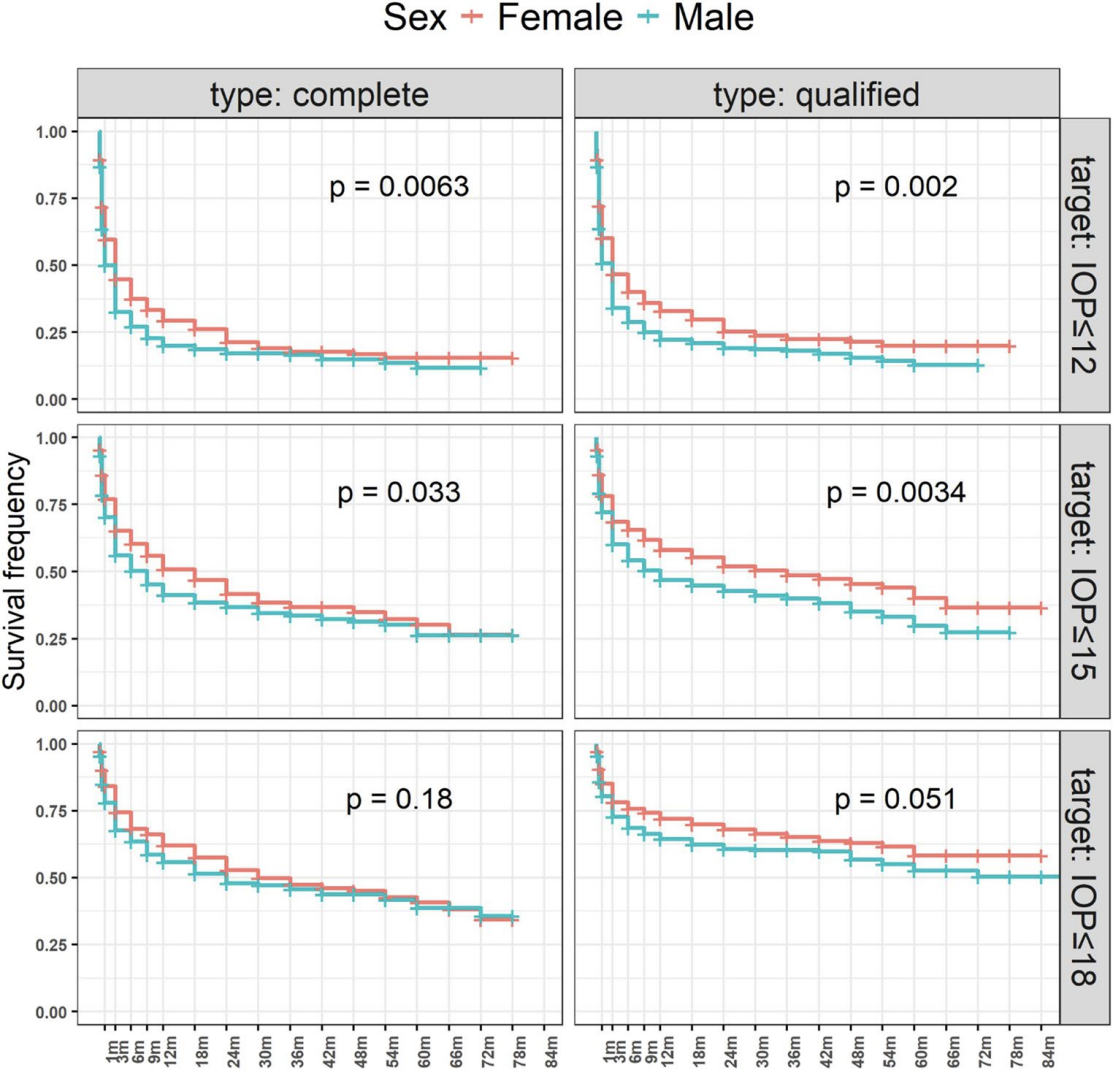
Success rates by gender. Complete success and qualified success are shown in the left and in the right graph, respectively. The Kaplan Meier survival curves of eyes from female patients are depicted in red, those from male patients in blue

The Kaplan Meier curves for male and female gender are shown in Fig. 2.

### Age

In the multivariable Cox regression analysis, age did not significantly influence complete or qualified success with target IOP level of ≤ 12 mmHg, ≤ 15 mmHg or ≤ 18 mmHg (*p* > 0.05, respectively).

### Baseline IOP

For a target pressure of ≤ 18 mmHg, complete and qualified success were significantly higher with higher baseline IOP (*p* = 0.007 for complete success, *p* < 0.001 for qualified success).

In contrast, the success rates at target IOP of ≤ 15 mmHg and ≤ 12 mmHg were not significantly dependent on baseline IOP (*p* > 0.05, respectively).

### Low early postoperative IOP

The strongest predictor of complete and qualified success was IOP on the first postoperative day. There were significantly higher complete and qualified success rates for all target pressure levels when IOP on the first postoperative day measured 0 to 4 mmHg compared to IOP values between 4 and 10 mmHg (*p* < 0.0001 each), indicating that a lower IOP on day one correlates with improved long-term success.

**Fig. 3:**
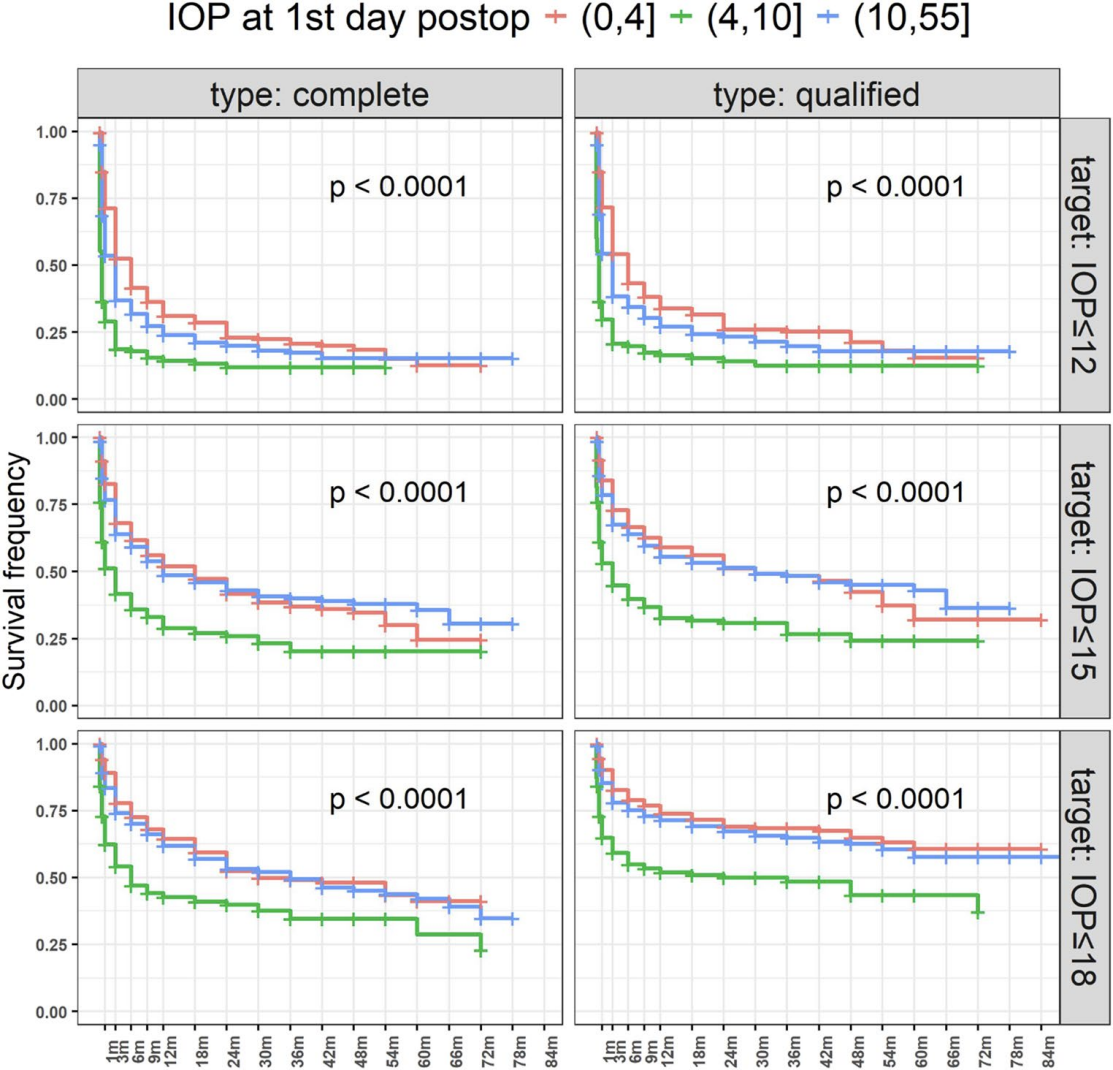
Success rates by postoperative day 1 IOP. Lower IOP levels on the first postoperative day were found to strongly predict higher success rates

The Kaplan Meier curves according to the level of the first postoperative day IOP are illustrated in Fig. 3.

### Antimetabolites

In the majority of cases (582 eyes, 75.3%), 25 µL of mitomycin C (MMC; 0.2 mg/mL, resulting in 5 µg of MMC) were injected into the subconjunctival space at 5 mm limbal distance superiorly. In 51 cases (6.6%), 50 µL (10 µg) of MMC was used, and in 114 eyes (14.7%), 100 µL (20 µg) of MMC was applied. In 26 cases (3.4%), 100 µL of 5-fluorouracil (5-FU; 50 mg/mL, resulting in 5 mg 5-FU) was applied instead.

Some MMC doses significantly influenced complete and qualified success in the multivariable Cox regression analysis. Pairwise comparison of different antimetabolite doses (5, 10 and 20 µg MMC, or 5 mg 5-FU) revealed significantly higher success with 5 µg MMC compared to 10 µg MMC with target IOP levels ≤ 12 mmHg (complete: *p* = 0.019, qualified: *p* = 0.012) and ≤ 15 mmHg (complete: *p* = 0.041, qualified: *p* = 0.012), but not with target IOP ≤ 18 mmHg (*p* > 0.05). Also, 5 µg MMC achieved significantly higher success with IOP ≤ 12 mmHg when compared to 5-FU (complete: *p* = 0.003, qualified: *p* = 0.001). However, no differences were found between the other concentrations of MMC or between MMC and 5-FU at the other target IOP levels (*p* > 0.05).

### Pseudophakia

 The multivariable Cox regression analysis revealed no significant influence of lens status (pseudophakic/phakic) on complete and qualified success rates across all target IOP levels (*p* > 0.05).

### Previous incisional glaucoma surgery

The complete success rate for the IOP target ≤ 18 mmHg was significantly higher in eyes without previous incisional glaucoma surgery (*p* = 0.02). The qualified success rate and all other success rates at the lower target IOP levels showed no significant differences between naïve eyes and eyes with previous incisional surgery (*p* > 0.05, respectively).

### Glaucoma subtype

Multivariable Cox regression analysis revealed no significant differences in surgical success depending on the subtype of glaucoma across all IOP target levels (*p* > 0.05, respectively).

## Discussion

To our knowledge, this is the first study to provide a comprehensive analysis of long-term success rates up to six years for the XEN gel stent while specifically examining the impact of demographic and clinical factors, on achieving varying IOP targets. Over a follow-up period of up to six years, the XEN gel stent demonstrated a consistent reduction in IOP, in line with previous large-scale registry data showing sustained efficacy over two years [5], while surgical success was influenced by factors such as sex, lower antimetabolite dose, and perioperative IOP.

Interestingly, sex impacts success rates, with female patients demonstrating higher complete and qualified success. These findings align with the results of Gillmann et al., who identified male sex as a significant risk factor for failure of XEN gel stent [6]. Although findings are not consistent across the literature, retrospective analyses have identified male gender as a potential risk factor for reduced success rates after trabeculectomy [7]. Also, in uveitic glaucoma, higher success rates following trabeculectomy have been reported in female patients [8]. Tan et al. identified male sex as a risk factor for surgical failure following combined phacoemulsification and excisional goniotomy in angle-closure glaucoma [9]. Potential differences in conjunctival wound healing between sexes may be a possible explanation for these findings. Coco et al. reported prolonged wound healing in females in a cohort with persistent corneal epithelial defects [10]. In contrast, Wagner et al. did not find any association between success rates and sex at 1 year after XEN gel stent implantation in a retrospective cohort study of 171 eyes [11], which might be attributed to the shorter follow-up period not fully capturing the long-term impact of sex on surgical success. Further research is warranted to examine whether sex-dependent wound healing dynamics impact surgical success rates after XEN gel stent implantation.

In our analysis, age did not significantly influence surgical success. This contrasts with experimental studies in murine models suggesting that with increasing age, inflammatory cells infiltrate more slowly, and TGF-β expression is reduced, potentially modulating the intensity of the scarring response [12–14].

Fibrotic processes, such as those driven by overproduction of extracellular matrix proteins and TGF-β induction, are known to play a central role in determining surgical outcomes in subconjunctival glaucoma procedures [15]. However, the impact of age-related extracellular matrix changes on wound healing in human ocular tissues remains insufficiently researched [16].

Regarding trabeculectomy, the impact of patient age on surgical outcomes remains controversial. Briggs and Jay found no significant difference in success rates across age groups [17], whereas Chiu et al. reported improved outcomes in patients over 70 years of age [18]. On the other hand, another large retrospective analysis suggests higher success rates after trabeculectomy in younger individuals [19]. These discrepancies may reflect differences in patient selection, surgical technique, or follow-up duration across studies.

In our study, a higher baseline IOP was associated with higher long-term success, compared to lower preoperative IOP levels. A recent multicenter, prospective study reported a low preoperative IOP < 15 mmHg as a factor associated with surgical failure [20]. A strong predictor of long-term success was IOP on the first postoperative day. Lower initial postoperative IOP might minimize early wound healing that contributes to bleb fibrosis. This aligns with findings by Mansouri et al., indicating that lower IOP on the first postoperative day was significantly associated with better surgical success at 12 months in POAG and pseudoexfoliative glaucoma (PXG) eyes [6, 21]. This implies potential prognostic value of pre- and postoperative IOP values for long-term success and might influence patient selection.

Different concentrations of MMC or 5-FU were used intraoperatively across cases. Our current analysis revealed superiority of 5 µg MMC compared to other doses and 5-FU for lower target IOP levels. However, a previous study from our group with a smaller study population of 54 eyes found no significant association between MMC concentration and surgical success at 24 months following XEN gel stent implantation [22]. Experimental in vitro studies demonstrated dose-dependent intrascleral MMC concentration [23] and apoptosis of fibroblasts of the Tenon’s capsule after MMC and high-dose 5-FU application [24]. However, a retrospective study showed no difference between 10 and 20 µg of MMC in 63 eyes after 12 months [25]. To our best knowledge, no previous study with a comparably large sample size has examined the influence of different antimetabolite doses. Further studies are warranted to verify dose-dependent differences in success.

Lens status was not significantly associated with surgical success in our cohort. In contrast, a previous study reported higher surgical success rates in pseudophakic eyes, albeit with a shorter follow-up period [26]. Evidence from other glaucoma procedures is mixed. A prospective clinical cohort study in Japan has shown trabeculectomy to be less successful in pseudophakic than in phakic eyes [27, 28]. Gedde et al. emphasize that tube shunt surgery is more promising than trabeculectomy for pseudophakic eyes, as demonstrated in prospective randomized and non-randomized studies [28, 29]. These heterogenous findings suggest that the influence of lens status likely varies across procedures and study populations. However, we did not observe any effect of lens status on success in our cohort.

Prior incisional glaucoma surgeries did not significantly affect success rates. These results are consistent with the findings of other studies, showing no difference in complete and qualified success of the XEN gel stent between naïve eyes and eyes with previous glaucoma surgery [30–32]. However, one study emphasized that a final IOP ≤ 18 mmHg was achieved more frequently in eyes without previous glaucoma surgery [30]. A retrospective case-control study comparing XEN gel stent with cyclophotocoagulation and traditional glaucoma drainage device in preoperated eyes demonstrated noninferiority of the XEN regarding IOP reduction and failure. However, an ab externo implantation route was chosen in that trial [33]. Our findings suggest that the XEN gel stent remains an effective option also in pre-operated eyes.

In our study, no significant differences in surgical success were observed between glaucoma subtypes. Although previous studies suggested higher success rates in PXG compared with POAG, our results did not confirm these differences. Rauchegger et al. reported higher complete success rates at 24 months in PXG compared to POAG, whereas the rate of qualified success was lower in PXG [34]. A prospective analysis by Gillmann et al. also observed higher success rates after 2 years with a target IOP of ≤ 16 mmHg for eyes with PXG than for those with POAG. However, XEN implantation was combined with cataract surgery in that study [35]. On the other hand, the absence of significant subtype-specific differences in our cohort suggests that the XEN gel stent may provide comparable efficacy across various glaucoma types.

A temporary decrease in mean BCVA at 12 months might be attributable to eyes with pre-existing retinal or glaucomatous comorbidities with fluctuating visual acuity during follow-up. No surgery-related cause for the transient decline was identified, and BCVA remained comparable to baseline at all subsequent visits.

This study shows that various factors influence the long-term success of the XEN gel stent, supported by a large sample size and a follow-up period of up to six years.

As a retrospective analysis, the study is subject to inherent biases, including selection and reporting biases. Prospective, randomized controlled trials to validate the identified success predictors are desirable for the future. Investigating the biological mechanisms behind the influence of these factors could help to optimize outcomes.

An additional limitation is the bilateral eye inclusion that may introduce inter-eye correlation and limit full statistical independence. Furthermore, some rare glaucoma subtypes were represented by very small groups, reducing statistical power for subgroup analysis. However, these cases were intentionally included to preserve the real-world nature of the dataset, increasing external validity.

## Conclusion

This study demonstrates the influence of multiple factors on long-term success following XEN gel stent implantation. Significant predictors of higher success rates included female sex, low IOP levels on the first postoperative day, moderately elevated preoperative IOP, and lower MMC doses, showing the importance of patient-specific and clinical factors in surgical outcomes.

The procedure achieved sustained reductions in intraocular pressure over a follow-up period up to six years. These findings reaffirm the utility of the XEN gel stent as an effective option for glaucoma management, particularly in challenging cases with varied clinical backgrounds.

## Data Availability

No datasets were generated or analysed during the current study.
